# Reflecting on philosophies of medical education science

**DOI:** 10.3205/zma001804

**Published:** 2026-01-15

**Authors:** Rachel Ellaway

**Affiliations:** 1 University of Calgary, Cumming School of Medicine, Office of Health and Medical Education Scholarship, Community Health Sciences, Calgary (AB), Canada

**Keywords:** philosophy, medical education, medical education research, philosophy of science

## Abstract

Science is dependent on philosophy for many of its core concepts, for its ongoing development, and for the means to appraise what it does and what it produces. Despite this, medical education scholars often seem to have little grounding in philosophy as it applies to the work they do. In this commentary, the author considers some key philosophical questions related to medical education. For example, what is the purpose of medical education? What are the meanings, uses, and implications of generalizability and the middle-range in medical education? How are theory and practice connected? What are the ontological, epistemological, and axiological capabilities of different theories and methodologies? In what ways and to what ends should knowledge claims be appraised? What are the limitations of science and the knowledge it can produce? What makes a ‘good’ researcher? Medical education scholars need to be able to answer these kinds of philosophical questions if the quality and utility of medical education science is to be assured.

## Abstract (de)

Wissenschaft ist in Bezug auf viele ihrer Kernkonzepte, ihre kontinuierliche Weiterentwicklung und die Mittel zur Bewertung ihrer Durchführung und Ergebnisse von der Philosophie abhängig. Dennoch scheinen Wissenschaftler*innen in der medizinischen Ausbildungsforschung kaum über philosophische Kenntnisse zu verfügen, die für ihre Arbeit relevant wären. In diesem Kommentar geht die Autorin auf einige zentrale philosophische Fragen im Zusammenhang mit der medizinischen Ausbildungsforschung ein. Was ist beispielsweise der Zweck der medizinischen Ausbildungsforschung? Welche Bedeutung, welchen Nutzen und welche Auswirkungen haben hier Verallgemeinerbarkeit und mittlere Reichweite? Wie sind Theorie und Praxis miteinander verbunden? Welche ontologischen, epistemologischen und axiologischen Potenziale weisen verschiedenen Theorien und Methodologien auf? Auf welche Weise und zu welchem Zweck sollten Erkenntnisansprüche bewertet werden? Was sind die Grenzen der Wissenschaft und der Erkenntnisse, die sie hervorbringen kann? Was macht eine*n „gute*n“ Forscher*in aus? Forschende im Bereich der medizinischen Ausbildung müssen in der Lage sein, diese Art von philosophischen Fragen zu beantworten, wenn die Qualität und der Nutzen der medizinischen Ausbildungsforschung gesichert werden soll.

## Overview

What we call “science” today was for a long time called “natural philosophy”, as it applied philosophical techniques of observation, analysis, and reasoning to the exploration of natural phenomena. Although they are now generally considered to be separate practices, science and philosophy are still deeply entwined. This is reflected in the ongoing dependence of science on philosophy to articulate its ideas, to justify its claims, and to change, adapt, and improve its practices. In this commentary, I invite medical education researchers to consider the many philosophical issues of medical education research and with those of the social sciences more generally. I would note however that, given the brevity of this format, the range of topics consider is limited and should be understood therefore as no more than a selective sample of the many intersections of philosophy with the sciences of medical education.

## Philosophy in medical education

Although there are many philosophical issues in medical education, they often go unacknowledged and unaddressed. For instance, each of us has ideas and beliefs as to what medical education is for, what assessment and feedback are, and what is meant by ethics and professionalism, but do we really understand them, do we agree on them, and is agreement even required? The plurality and divergence of definitions and uses of these terms would suggest that while agreement is elusive it does not undermine the ability of scholars to produce valuable research. 

Philosophy is also reflected in the meanings and purposes we perceive or attribute to the things we do. For instance, is the purpose of medical education to control who gets into the profession, or is it about the transfer of knowledge and experience in preparation for practice? Is the purpose of medical schools to socialize learners to a profession, to produce a workforce, or to meet a social contract? Should medical teachers focus on minds, persons, classes, cohorts, or whole programs? Should medical education researchers describe or clarify current practices, or challenge them or seek to change them? Is the purpose of program evaluation to maintain standards (quality assurance) or to drive innovation and problem-solving (quality improvement)? 

Note that I have presented these arguments as a series of false dilemmas; the options I suggest are not the only way of answering the questions I pose. A good philosopher might be expected to be able to recognize this and argue that there may be other factors to consider, that perhaps none of the proffered options is correct, and that many factors may interact such that answers are not easily distinguishable. Indeed, without a focus on philosophical matters, much of what we do is based on unexamined assumptions, values, and beliefs.

## Generalizability, naturalism, and the middle-range

The natural sciences, particularly those of physics and chemistry, are often held to be the ideal for all scientific inquiry, and by comparison, the social sciences have often been considered to be weak and ineffective [1]. Medical education is a part of the social sciences and shares many of its intrinsic challenges, including how to accommodate contextual entanglements, voluntarism, and complexity. To that end, the sciences of medical education are intrinsically “middle range”, a concept advanced by Merton [2] to reflect theory that can only generalize to some extent. There are reflections of this in the use of the concept of “transferability” (instead of “generalizability”) in many qualitative methodologies, and in the statistical focus on generalization to a specified population rather than to all possible populations. The differences between naturalistic and interpretivist philosophies in the social sciences are not only realized in the practicalities of inquiry, they also reflect the beliefs and goals of the scientists designing, conducting, and reporting acts of inquiry. For instance, scientific naturalists believe in the possibility that general (if not universal) laws of human behaviour can be found or developed, while scientific interpretivists believe that human behaviour can only ever be interpreted and not reduced to laws or algorithms [3]. As much as the differences between qualitative and quantitative philosophies of inquiry are taken to be the primary bifurcation of medical education sciences, I would suggest that the naturalist-interpretivist divide is the more fundamental of the two, even though it is largely unacknowledged.

## Theory and practice

**Figure 1:**
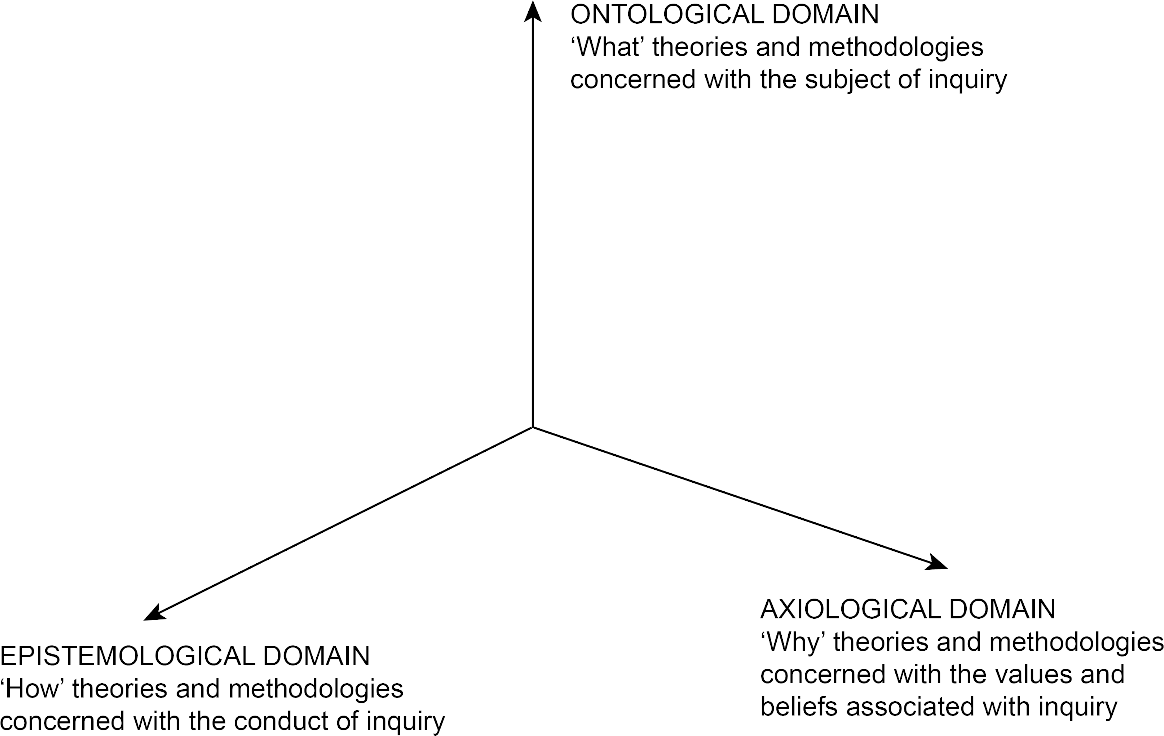
The three domains of theory and methodology. Typically using a theory or methodology from any one domain cannot address the issues and questions associated with other domains. Robust inquiry depends on combining theories and methodologies to be able to cover all three domains.

The maxim “theory without experience is empty, but experience without theory is blind” is attributed (erroneously) to Prussian philosopher Immanuel Kant, the point being that neither empiricism (action and evidence) nor theory (reflection and abstraction) can exist meaningfully without the other. The principle that theory guides action and action shapes theory is generally accepted … except that not all actions and theories are equivalent. Clearly, medical education sciences encompass divergent methodologies that afford very different kinds of knowledge and understanding. Some theories focus on what is being inquired into, some theories focus on how inquiry should be conducted, and some theories focus on values in inquiry. For instance, phenomenological theories are largely focused on the nature of human experience but have relatively little to say about how to explore it or why. Grounded theory (in its various guises) and the various theories of statistics focus on the “how to” of inquiry but are quite agnostic as to the “what” or “why”. Critical theories meanwhile (and other normative theoretical positions) tend to be focused much more on the “why” than on the “what” or “how” of inquiry [4]. Given that good research involves attention to all three dimensions, can a single theoretical or methodological achieve this? I would argue that scientists need to combine theories and methodologies to ensure all three axes are covered, see figure 1 (Fig. 1). 

## Phenomena and noumena

Social scientists often work with abstract and latent constructs. While the natural sciences also use latent constructs, such as in high-energy physics, these constructs are taken to be real but unobservable (like dark matter, different nuclear forces, and gravitons). In the social sciences latent constructs are often understood to be social conventions and categories, such as professionalism, competence, vocation, and identity. We can attribute observable, measurable non-latent constructs to latent constructs, but the construct itself is still a social convention. This reflects a Kantian separation of phenomena (things we can directly observe i.e. through empiricism) and noumena (things we cannot directly observe). That said, the issue is not just a matter of distinguishing between phenomena and noumena, we also need to consider whether the translation between non-latent and latent variables and constructs is logical, reliable, and credible. Given the importance we tend to assign to noumena (such as competence, professionalism, and fitness to practice) medical education scientists need to be able to distinguish between phenomena and noumena, to understand the theoretical connections between them, and appraise the knowledge claims that can be made about phenomena and noumena whatever their paradigmatic allegiances. 

## Knowledge claims

Knowledge claims are assertions of truth that broadly take one of five forms. An inductive knowledge claim is supported by empirical evidence, such as the findings of an empirical study. A deductive knowledge claim is supported by a priori knowledge, such as translating theory to a study design. An abductive knowledge claim is based on a reasoned argument, such as logically identifying likely causes of a phenomenon. A referred knowledge claim is supported by the work of other scholars (in which case that work is cited and referenced). There are also knowledge claims that are made with no supporting evidence, reasoning, or precedent. The strength of the knowledge claim being made should be proportionate to the evidence or supporting argument. Knowledge claims that overstate, misinterpret, or misappropriate whatever is presented to back them up are unacceptable. Much of science involves developing, testing, and using knowledge claims. Critical appraisal involves testing and evaluating the knowledge claims made in scientific communications (papers, presentations, grant applications). Unfortunately, this testing of knowledge claims does not seem to be the norm in medical education and as a result there is much that is published or presented that makes weak or flawed knowledge claims.

## Limitations of science

Following on from my last point, if much of what is published in our field is opinion-based, does this mean that the medical education literature is unscientific? For example, this paper is not empirical or otherwise based on the ‘scientific method’. However, I would argue that this paper and many other non-empirical texts reflect defensible standards of metascholarship in that they present both argument and evidence in support of abductive knowledge claims such that the strengths and weaknesses of these knowledge claims can be scrutinized. 

In an applied field, there is often a pragmatic sense that all reliable knowledge is (or should be) useful knowledge and the markers of quality reflect standards of defensibility and utility. This can be countered by a naturalistic argument that good science should not include nonempirical knowledge and should only be based on high quality methodological designs. Rather than playing the somewhat futile game of ‘whose paradigm is better?’, we can again draw on the philosophy of science to consider the strengths and limitations of the science in our field. Karl Popper argued that a theory is scientific only if it is falsifiable by empirical means, and that science only advances through the falsification of theory [5]. Popper was challenged with a counterargument that, if all theories are approximate, then what is the value of science? Popper countered this by arguing that, although science is imperfect, the only way to improve it is to pursue it with an understanding that the knowledge it produces is at best an approximation to the truths that it pursues. All those involved with medical education science should understand that the science we do is inescapably imperfect and yet utterly necessary if our understanding is ever to be improved or expanded. They should also understand that much of the medical education knowledgebase is based on reasoned argument rather than solely on empirical findings.

## What makes a “good” researcher?

Building on the Popperian understanding of the limitations of science, the last question I will explore is “what constitutes a good researcher?”. Much philosophical thought has been focused on what makes a good person or a life well-lived, and similar arguments might be applied to what makes a good researcher. One philosophical line of argument might reflect a “virtue ethics” perspective that focuses on individual virtue and character, and qualities of responsible inquiry such as honesty, objectivity, integrity, courage, respect for others, openness, and accountability. A utilitarian line of argument might focus on the value of a researcher’s contributions with particular attention paid to outcomes, consequences, and harms. A deontological approach would focus on ideals such as duties, rights, justice, and fairness, and how well they were reflected in a researcher’s work. Although each of these approaches can answer the question of “what makes a good researcher?” they start from different assumptions and positions and will likely come to different conclusions. Rather than saying that any one approach is superior, we might instead understand them to reflect different kinds of evidence that create a holistic understanding of the quality of a researcher. That such questions cannot be answered by measurement but require interpretation and judgment is something we see in much of medical education. It is in the validity evidence theories of Kane [6], it is in many models of medical school admissions, and it is central to the aggregative philosophy of programmatic assessment [7]. This in turn reinforces a need for attention to matters of axiology (belief, value, ethics, esthetics) as well as to ontology (what exists, similarity and difference) and epistemology (knowledge and understanding).

## Closing thoughts

I have argued that philosophy provides the conceptual and procedural foundation for the sciences of medical education. Philosophy adds depth, direction, and meaning. It allows us to see science as it is, not as it is believed to be. It allows science to be critiqued, to be challenged and defended. Defensibility is, after all, the default and broadly accepted standard for quality in academic work. That said, can we defend the philosophical basis of medical education science? Do we even try? Tacet philosophy is everywhere in med-ed science, and yet there is apparently little awareness of this, and little competence, confidence, or interest in philosophical matters. This seems particularly ironic in a field that has both doctors of philosophy and doctors of medicine but that does not seem to require basic philosophical competence of either. If we are to improve and develop the sciences of medical education, if we are to improve critical and logical thinking and analysis, if we are to be more realistic and grounded in what we do and the knowledge claims we make, if we are to both honour the traditions of science and to challenge them, then this requires philosophical competence of our scientists and scholars. I have sketched out a number of dimensions and issues in this commentary, but they are just a sample. There are many more aspects of philosophy to consider and work on in medical education. I will end by asking; what role does philosophy play in the sciences of medical education that you are involved in?

## Author’s ORCID

Rachel Ellaway: [0000-0002-3759-6624]

## Competing interests

The author declares that she has no competing interests.
